# Fluorescence-Only Readable Micro-QR Codes Achieved by Hydrogel Encapsulation

**DOI:** 10.1021/acsomega.6c03918

**Published:** 2026-06-26

**Authors:** Yongjae Song, Jaesung Park, Changhong Cao, Hyeli Kim, Dong Chan Kim, Cheolheon Park, Daewon Lee

**Affiliations:** † Department of Photonics and Nanoelectronics, 65354 Hanyang University, Ansan 15588, Korea; ‡ BK21 FOUR ERICA-ACE Center, Hanyang University, Ansan 15588, Korea; § Department of Mechanical Engineering, 5620 McGill University, Montreal, Quebec H3A 0C3, Canada; ∥ Bhome Gen Co., Ltd, Bucheon 14560, Korea; ⊥ Department of Chemical, Biological and Battery Engineering, 65440 Gachon University, Seongnam 13120, Korea; # Department of Semiconductor Engineering, Gachon University, Seongnam 13120, Korea; ∇ Department of Electronic Engineering, 26714 Jeonbuk National University, Jeonju 54896, Korea

## Abstract

Quick response (QR) codes are widely used for information storage and authentication; however, their macroscopic visibility and ease of replication limit their security performance in anticounterfeiting applications. Reducing QR patterns to the microscale can increase replication difficulty, yet the encoded structures remain optically detectable under conventional microscopy. In this study, we present a hydrogel encapsulation strategy that transforms microscale QR codes into covert tags that are indistinguishable under bright-field observation while remaining selectively readable through fluorescence imaging. Micro-QR patterns were fabricated using a photocurable hydrogel system and subsequently encapsulated within a secondary hydrogel matrix with distinct optical properties, resulting in effective suppression of visible contrast while preserving fluorescence signal transmission. The encapsulated structures exhibited no recognizable pattern under bright-field conditions while enabling reliable decoding under fluorescence microscopy. This approach combines optical masking and physical isolation to enhance resistance against visual inspection and direct replication, providing a simple yet effective platform for covert information encoding. The proposed hydrogel-based encapsulation strategy offers potential applications in anticounterfeiting, secure labeling, and microscale authentication technologies.

## Graphical abstract

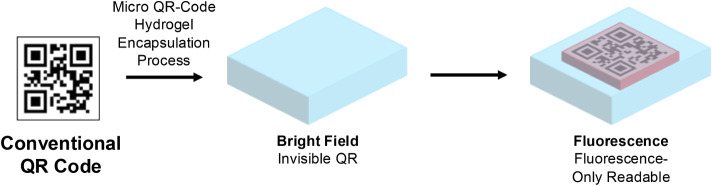

## Introduction

Anticounterfeiting and secure labeling technologies are increasingly important in a wide range of fields including pharmaceuticals,^1,2^ valuable products,^3^ and electronic components.^4,5^ To address the growing demand for authentication systems, various micro and nanoscale taggant technologies^6–10^ have been developed to encode identification information directly within materials or devices. Such taggants can function as physical security features that are difficult to replicate using conventional manufacturing or printing techniques. However, many existing taggant systems rely on optically visible patterns or structures that can still be directly observed, imaged, and potentially reproduced once detected.

To improve the security of physical taggant systems, recent efforts have focused on reducing the size of encoded patterns to the microscale.^6,11^ Microscale structures require specialized fabrication methods and high-resolution patterning^6,12–15^ which significantly increasing the difficulty of replication. As a result, microscale tags have been investigated as potential candidates for next-generation authentication platforms. However, even microscale patterns remain optically detectable under conventional microscopy, allowing the encoded information to be visually inspected and potentially reconstructed. Therefore, strategies that can simultaneously suppress optical detectability while preserving reliable readout of encoded information are highly desirable.

Hydrogel-based materials offer unique advantages for constructing such covert encoding platforms due to their tunable optical properties, mechanical flexibility, and compatibility with photopatterning techniques. Photocurable hydrogels^16–18^ have been widely employed in microfabrication processes to generate microscale structures with precise geometries. Furthermore, hydrogel matrices can be engineered to modulate optical contrast through control of refractive index and light scattering, enabling the possibility of concealing embedded structures from conventional bright-field observation while maintaining functionality in alternative optical modes.

Here, we demonstrate a hydrogel encapsulation strategy^9,19,20^ for converting microscale encoded patterns into covert information tags, remaining visually indistinguishable under bright-field observation while being selectively readable through fluorescence imaging. As a model system, microscale QR patterns were fabricated using a photocurable hydrogel and subsequently encapsulated within a secondary hydrogel matrix possessing distinct optical properties. The encapsulation process effectively suppresses visible pattern contrast while preserving fluorescence-based decoding capability. This approach integrates microscale encoding with optical masking through hydrogel encapsulation, providing a simple and scalable platform for covert taggant systems with potential applications in anticounterfeiting, secure labeling, and microscale authentication technologies.

### Fabrication Strategy for Fluorescence-Only Readable Micro-QR Codes

To fabricate covert microscale taggants that are invisible under bright-field observation while remaining readable under fluorescence imaging, a multilayer hydrogel fabrication strategy was developed using Optofluidic Maskless Lithography system (OFMLs)^9,12^ Conventional macroscopic QR codes are directly visible and easily replicated, thereby limiting their security performance (Figure 1a). Reducing QR code structures to the microscale increases fabrication complexity, and the residual optical visibility allows them to remain observable under conventional optical microscopy. (Figure 1b). To overcome this limitation, hydrogel encapsulation was introduced to convert the microscale QR pattern into an optically masked covert tag (Figure 1c). Representative microscopy images confirm that the encapsulated QR structure becomes indistinguishable under bright-field observation while remaining clearly readable under fluorescence imaging (Figure 1d).

**Figure 1:**
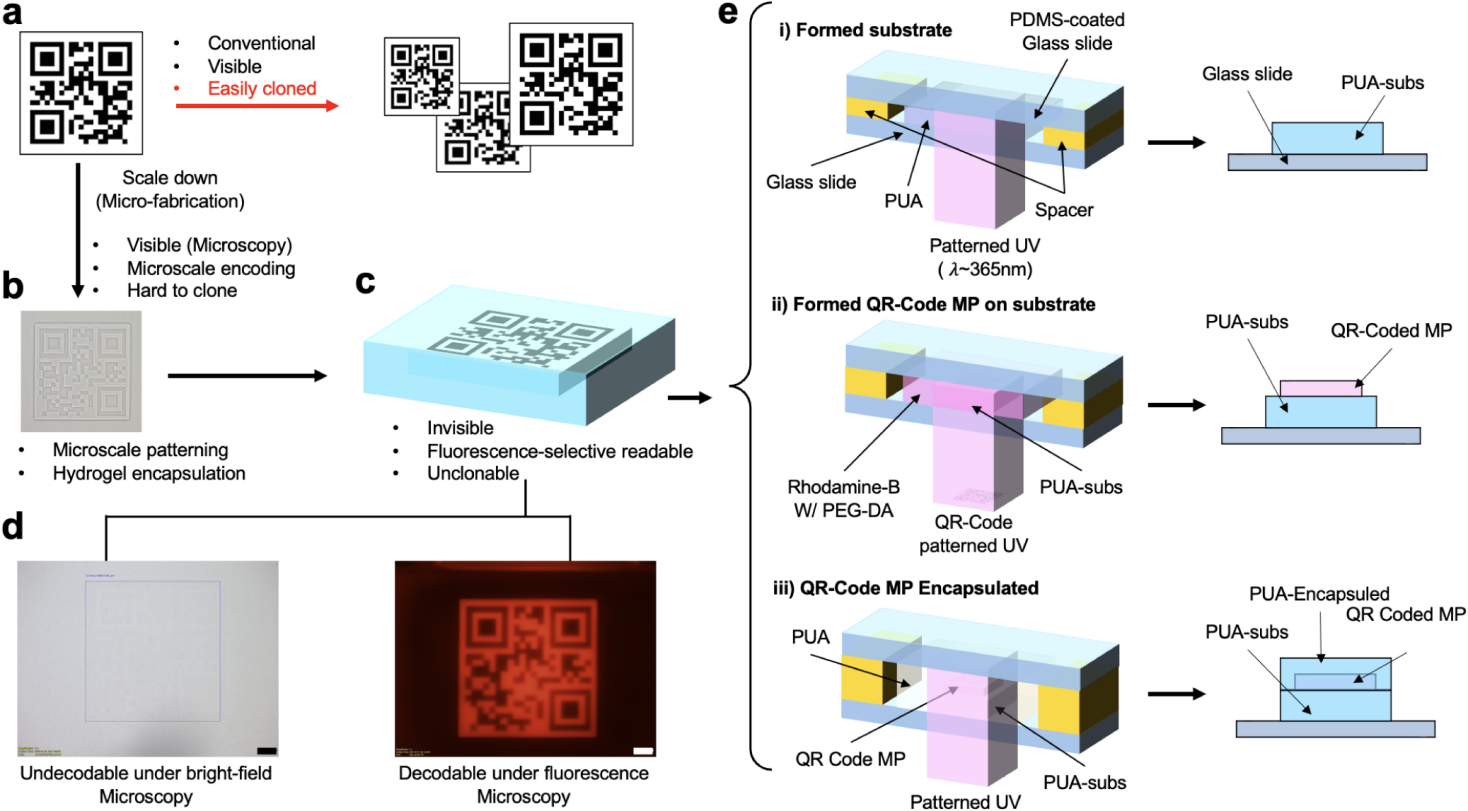
Concept and fabrication strategy of fluorescence-only readable micro-QR codes achieved by hydrogel encapsulation. (a) Conventional macroscopic QR codes are directly visible and readily readable under normal observation conditions. Because of their optical visibility, encoded information can be easily cloned or replicated. (b) Microscale reduction of QR structures increases fabrication difficulty, with the encoded pattern remaining optically detectable under microscopy. (c) Hydrogel encapsulation converts the microscale QR structure into an optically masked covert tag that is invisible under bright-field observation while remaining selectively readable under fluorescence excitation. (d) Representative microscopy images of the encapsulated micro-QR structure showing suppressed visibility under bright-field microscopy (left) and successful decoding under fluorescence microscopy (right). (e) Sequential OFML-based fabrication process of the multilayer micro-QR structure: (i) formation of the PUA substrate layer, (ii) selective photopolymerization of Rhodamine-B-doped PEGDA to generate the QR-coded micropattern, and (iii) formation of the upper PUA encapsulation layer. Scale bar: 100 μm.

Two photocurable materials were employed to construct the multilayer structure with polyurethane acrylate (PUA) and polyethylene glycol diacrylate (PEGDA). PUA was selected for structural layers due to its relatively high mechanical stiffness and low swelling property,^21–23^ while PEGDA was used as the encoding layer because of its optical transparency and compatibility with fluorescent dye incorporation.^23–25^ To generate fluorescence-readable encoding patterns, Rhodamine B was incorporated into the PEGDA precursor solution.^26^ The dye was dissolved in ethanol (1 mM) and subsequently mixed with PEGDA together with 1 wt % of the photoinitiator Irgacure 1173.

The fabricated micro-QR tag consists of a three-layer architecture comprising a PUA substrate, a fluorescent PEGDA micro-QR layer, and a PUA ceiling layer. The bottom PUA substrate provides mechanical support for the patterned microstructure, while the top PUA layer serves as an encapsulation layer that optically masks the encoded pattern under bright-field illumination. This encapsulation strategy plays a critical role in suppressing visible contrast of the QR structure while preserving fluorescence-based readability.

The fabrication process was performed sequentially using OFML-based photopolymerization (Figure 1e). First, the PUA substrate layer was patterned on a glass slide (Figure 1e (i)). Subsequently, Rhodamine-doped PEGDA was deposited and selectively polymerized using a QR-pattern mask to form the fluorescent micro-QR structure (Figure 1e (ii)). Finally, a PUA encapsulation layer was polymerized above the patterned QR layer to complete the covert tagging structure (Figure 1e (iii)). This multilayer configuration allows the encoded structure to be physically protected and optically concealed within the hydrogel matrix.

### Optical Masking and Fluorescence-Selective Decoding

The primary objective of the encapsulation strategy is to suppress optical visibility of the encoded microstructure under conventional bright-field observation while maintaining reliable fluorescence-based readout. Without encapsulation, microscale QR patterns fabricated from fluorescent PEGDA remain optically detectable under bright-field microscopy due to refractive index contrast between the patterned structures and the surrounding medium (Figure 2a (i)).

**Figure 2:**
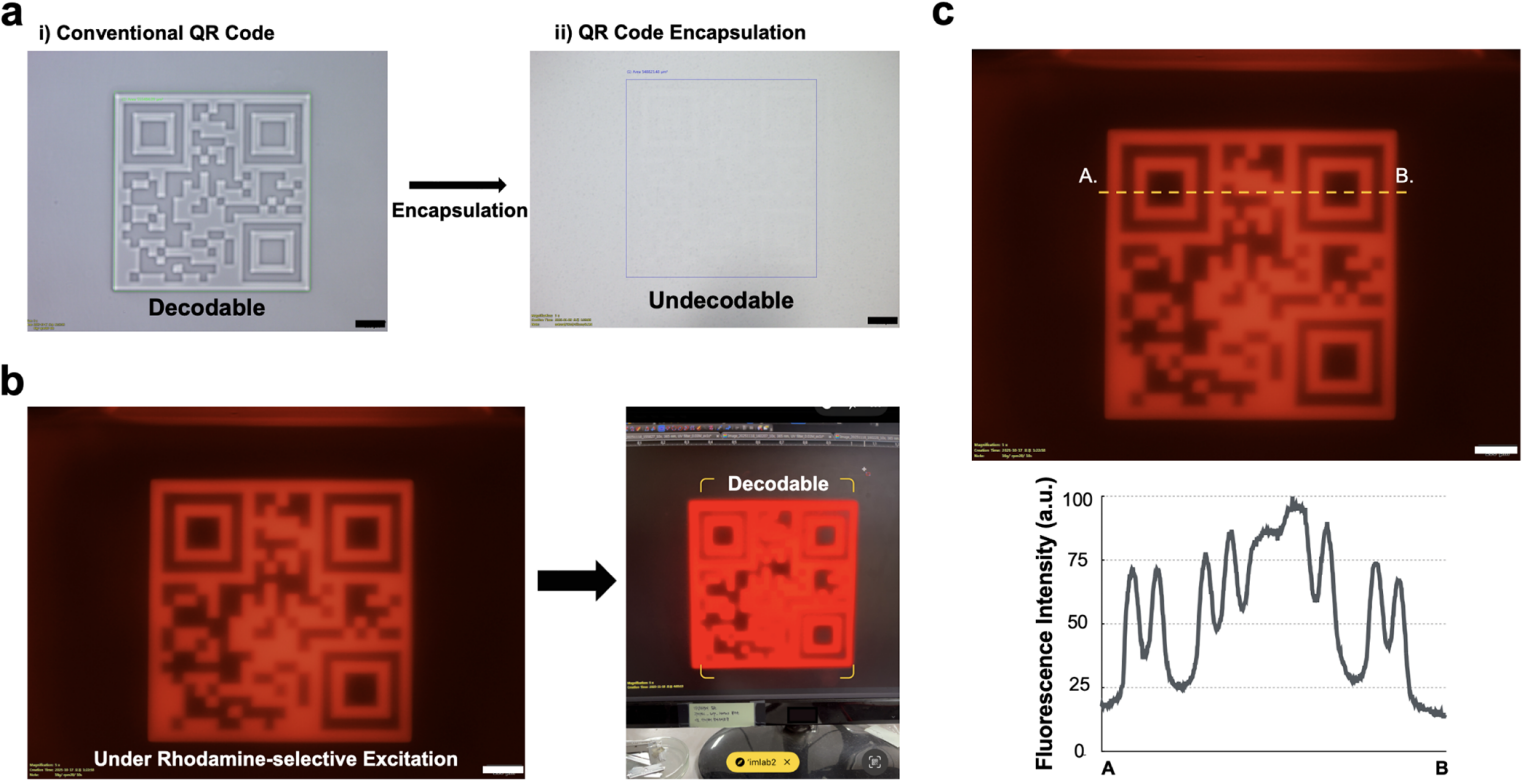
Optical masking and fluorescence-selective decoding of encapsulated micro-QR structures. (a) Bright-field microscopy images showing (i) a conventional fluorescent micro-QR pattern before encapsulation and (ii) an optically concealed structure after PUA encapsulation, where the encoded pattern becomes indistinguishable under bright-field observation. (b) Fluorescence image of the encapsulated micro-QR structure under Rhodamine-selective excitation and successful decoding using a smartphone QR reader. (c) Fluorescence intensity profile measured along the A–B direction across the encoded region, showing clear contrast between encoded and nonencoded areas under wavelength-selective observation. Scale bar: 100 μm.

After encapsulation with the PUA ceiling layer, however, the visible contrast of the QR pattern becomes significantly reduced. As a result, the encoded structure becomes indistinguishable under bright-field imaging conditions, appearing as a uniform hydrogel structure (Figure 2a (ii)). In contrast, fluorescence imaging selectively reveals the embedded QR pattern because the fluorescence signal originates only from the Rhodamine-containing PEGDA regions.

Fluorescence imaging was performed using an Olympus microscope (Samples were imaged using a Optical microscope (NFEC-2021-08-272555) at the Next Generation Display Research Core Facility Hanyang University ERICA) equipped with a U-MWG2 filter set. This filter configuration is suitable for Rhodamine B observation, with excitation wavelengths of 510–550 nm and emission detection around 590 nm. Under these conditions, the fluorescence micro-QR pattern was clearly observed and successfully decoded using a smartphone QR reader (Figure 2b).

To further evaluate the practical decoding reliability of the proposed system, statistical analyses were performed under varying viewing distances and observation angles. Stable decoding was achieved with a 100% success rate (20/20 trials) at distances up to 2.3 m and viewing angles up to 55°, demonstrating robust decoding performance over a broad observation range. Representative decoding images and quantitative success-rate analyses are provided in Figure S2.

To quantitatively evaluate the fluorescence contrast between encoded and nonencoded regions, fluorescence intensity profiles were analyzed using line profile measurements. The results confirmed a clear intensity difference corresponding to the island-structured QR pattern, which enables reliable decoding of the encoded information (Figure 2c).

To quantitatively evaluate the bright-field concealment effect of the encapsulation layer, gray-level intensity analysis was additionally performed using ImageJ. The results confirmed a significant reduction in optical contrast after encapsulation, quantitatively supporting the suppression of visible pattern recognition under bright-field conditions (Figure S3).

The geometrical parameters of the fabricated QR structures were further analyzed to evaluate the practical scaling behavior and information-encoding capability of the proposed system. The QR code used in this study corresponds to a standard Version 2 structure (25 × 25 modules), and the minimum feature size (module size) was estimated to be approximately 28.8 μm based on the design-defined module number. This estimation was based on optical microscopy images, although minor deviations may occur due to optical blur and signal diffusion during fluorescence imaging. Reliable decoding was achieved at the current size (∼720 μm), while further scaling toward smaller dimensions may require additional optimization of fabrication resolution and optical detection conditions, including signal-to-noise ratio and fluorescence intensity. In addition, reducing the QR-code size decreases the number of resolvable modules, which influences the achievable information capacity. These results provide insight into the relationship between structural miniaturization and information density in the proposed system.

### Multispectral Micro-QR Encoding

To further expand the information encoding capability of the system, multispectral micro-QR tags were fabricated by incorporating different fluorescent dyes within separate encoding layers. In addition to Rhodamine B, Coumarin 7 was introduced as a second fluorescent encoding material.

The Coumarin-based fluorescent precursor was prepared by mixing PEG-DA with Irgacure 1173 (5 wt %) and an acrylate-functionalized Coumarin 7 dye dissolved in ethanol. A higher concentration of photoinitiator and fluorescent dye was required because Coumarin 7 exhibits strong UV absorption during photopolymerization, which reduces polymerization efficiency and fluorescence intensity at lower concentrations.

The multispectral micro-QR structure consists of five layers: PUA substrate, Rhodamine-encoded QR layer, PUA middle layer, Coumarin-encoded QR layer, and PUA ceiling layer. The middle PUA layer physically separates the two fluorescent encoding layers, enabling independent optical observation of each QR code (Figure 3a).

**Figure 3:**
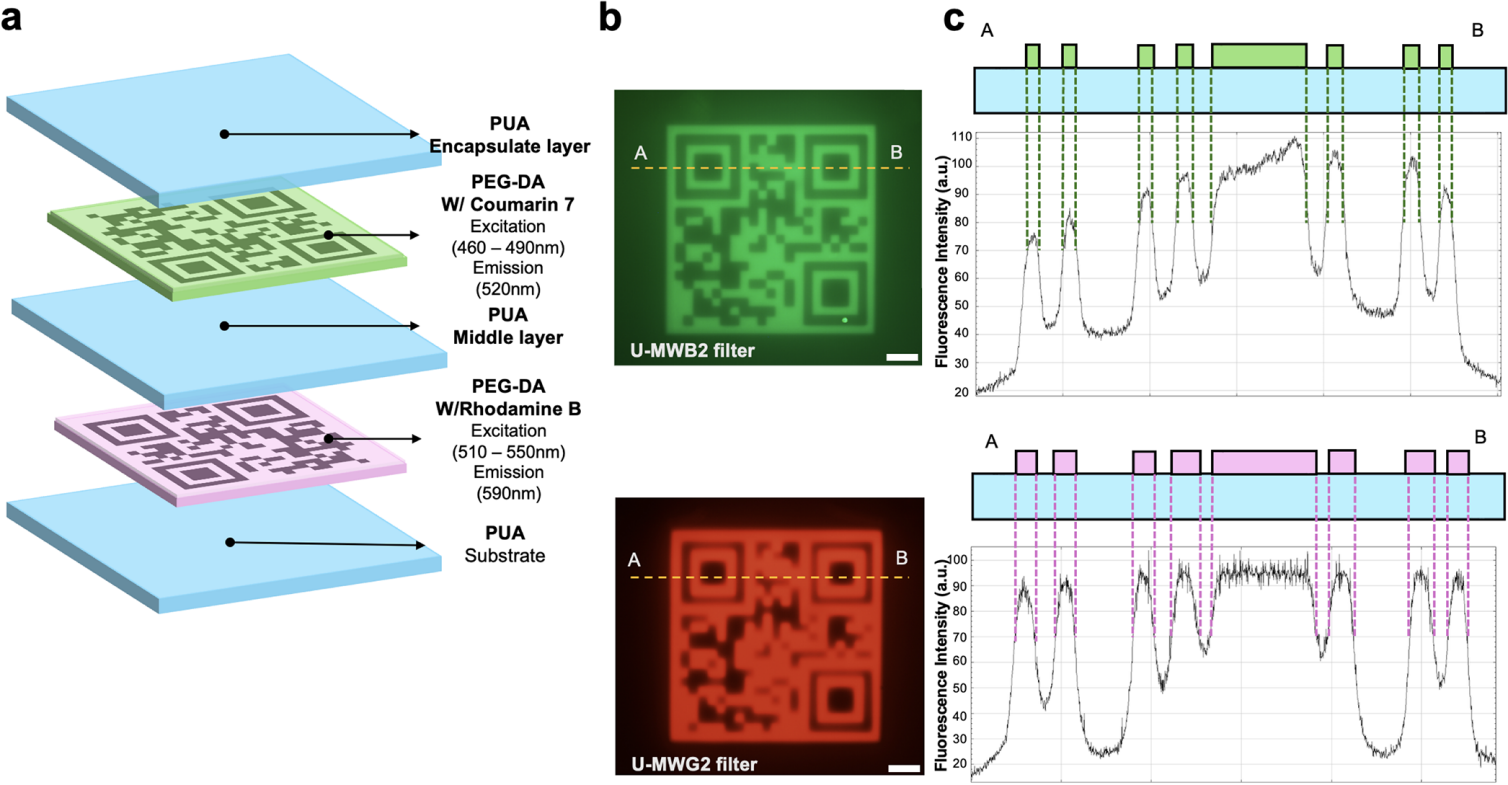
Multispectral hydrogel-encapsulated micro-QR structure for wavelength-selective fluorescence decoding. (a) Schematic illustration of the multilayer architecture consisting of a PUA substrate, Rhodamine B-encoded PEGDA layer, PUA middle layer, Coumarin 7-encoded PEGDA layer, and PUA encapsulation layer. (b) Selective fluorescence decoding of the embedded QR patterns under different filter conditions: Rhodamine-encoded layer observed under U-MWG2 filter and Coumarin-encoded layer observed under U-MWB2 filter. (c) Fluorescence intensity line profiles measured along the A–B direction, demonstrating wavelength-selective signal generation only under the corresponding fluorescence filter conditions. Scale bar: 100 μm.

Fluorescence imaging confirmed that the two QR patterns could be selectively observed using different filter sets. Rhodamine-encoded QR patterns were observed using the U-MWG2 filter, while Coumarin-encoded QR patterns were observed using the Olympus U-MWB2 filter (excitation 460–490 nm, emission 520 nm). Each QR pattern was successfully decoded under its corresponding fluorescence channel, while remaining undetectable under the other filter conditions (Figure 3b). These results demonstrate that multilayer hydrogel structures enable multiplexed covert information encoding (Figure 3c).

In addition, the predefined excitation/emission wavelength conditions may themselves function as an additional authentication factor. Since the encoded QR information can only be selectively decoded under specific optical conditions, the wavelength information may effectively serve as an authentication key for secure access to the embedded information. The multilayer multispectral QR-code architecture additionally enables hierarchical information encoding within a single microparticle platform. Different QR codes can independently store distinct levels of information, such as general identification data and higher-security authentication data, which are selectively accessible under predefined fluorescence wavelength conditions. In addition, simultaneous decoding of multiple wavelength-selective QR codes may provide an additional authentication layer for secure information access. Such multispectral fluorescence encoding expands the functionality of conventional single-channel QR taggant systems and provides enhanced flexibility for covert anticounterfeiting applications.

### Demonstration of Covert Tagging on Practical Substrates

To evaluate the applicability of the fabricated micro-QR tags as practical taggant systems, the structures were detached from the fabrication substrate and integrated into various objects, including glass wafers and pharmaceutical tablets (Figure 4a, b).

**Figure 4:**
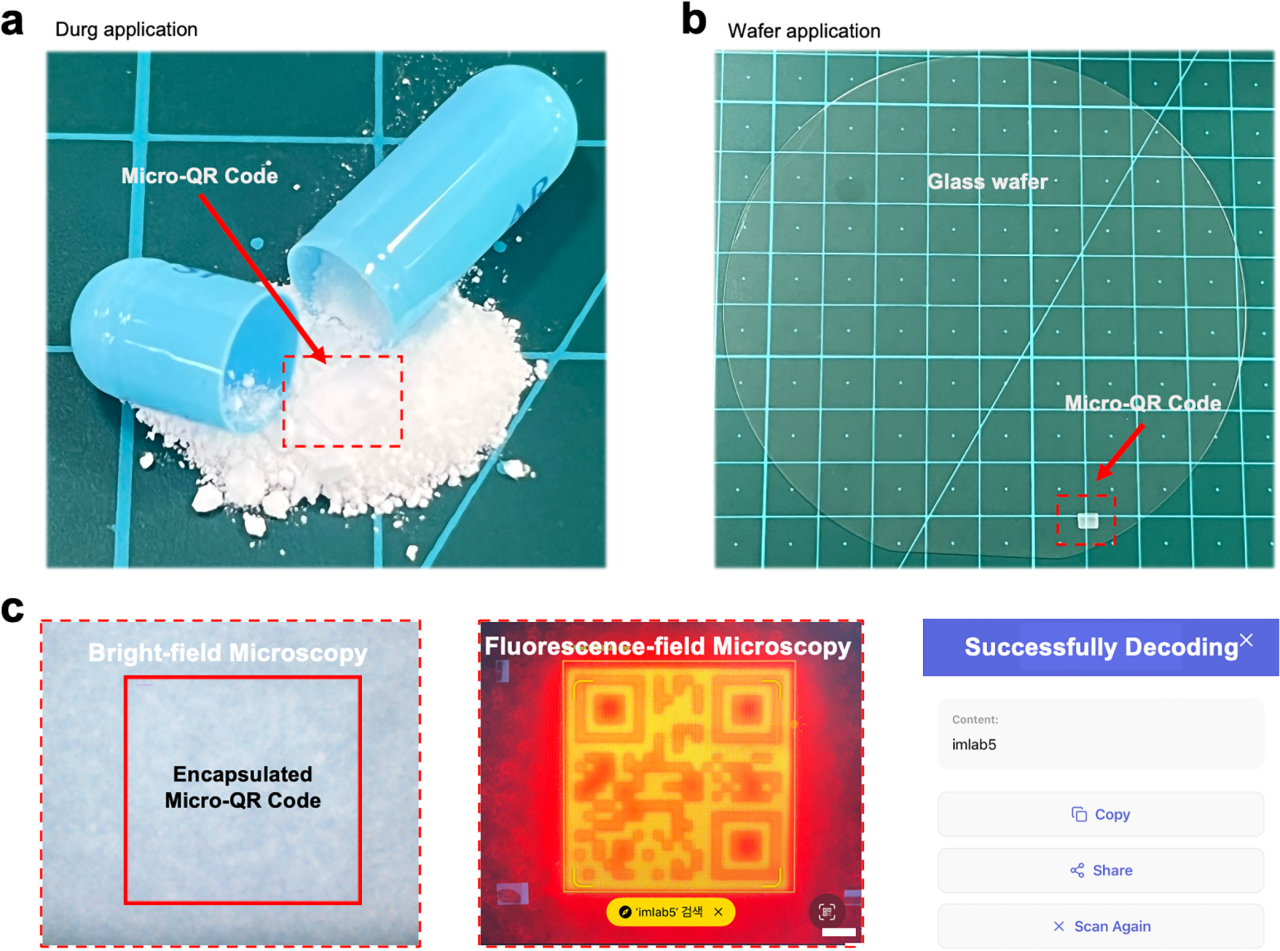
Application and fluorescence-based decoding of encapsulated micro-QR codes. (a) Demonstration of micro-QR code application in a pharmaceutical context. The encapsulated micro-QR code particles are embedded within powder inside a capsule, indicating potential use for anticounterfeiting and authentication of drug products. (b) Demonstration of micro-QR code integration on a glass wafer substrate. The micro-QR code particles are distributed on the wafer surface, suggesting applicability in semiconductor or device-level tagging and traceability. (c) Optical and fluorescence imaging results of the encapsulated micro-QR code. Under bright-field microscopy, the micro-QR code remains indistinguishable due to optical masking by encapsulation. In contrast, under fluorescence microscopy using a U-MWG2 filter set (Excitation: 510–550 nm, Emission: ∼590 nm), the embedded QR pattern is clearly visualized and successfully decoded using a smartphone-based reader, confirming selective fluorescence-based readability. Scale bar: 100 μm.

Under bright-field observation, the embedded micro-QR tags were visually indistinguishable from the surrounding material, confirming the effectiveness of the optical masking strategy. However, when observed under the appropriate fluorescence excitation conditions, the encoded QR patterns became clearly visible and could be successfully decoded (Figure 4c).

These demonstrations indicate that the encapsulated micro-QR structures can function as covert identification tags remaining hidden during normal inspection and becoming readable under controlled optical conditions. Such properties may provide potential applications in anticounterfeiting, secure labeling, and microscale authentication systems.

### Environmental Stability under Harsh Damp-Heat Conditions

For practical anticounterfeiting and authentication applications, environmental stability of the encapsulated fluorescent QR-code structures is an important consideration. Encapsulation-based microsystems have previously been investigated for protecting microscale coded structures and biologically sensitive materials from external environmental conditions.^27^ In addition, harsh damp-heat conditions such as 85 °C and 85% relative humidity (RH) are widely used accelerated reliability-testing conditions for evaluating environmental stability under severe thermal and moisture stress.^28^ Therefore, damp-heat aging tests were performed at 85 °C/85% RH to evaluate the environmental stability of the proposed encapsulated fluorescence QR-code system. Time-dependent fluorescence imaging, fluorescence line-profile analysis, and fluorescence contrast evaluation were conducted during the damp-heat aging process.

As shown in Figure 5a, gradual fluorescence blurring and reduced pattern distinguishability were observed with increasing damp-heat aging time. Corresponding fluorescence line-profile analysis demonstrated progressive reduction in peak-to-valley intensity difference, indicating decreased fluorescence contrast and fluorescence distinguishability over time (Figure 5b). Quantitative fluorescence contrast analysis additionally confirmed gradual reduction of the fluorescence contrast ratio during prolonged damp-heat aging (Figure 5c). These results suggest that prolonged damp-heat aging gradually reduces fluorescence contrast between the QR-coded regions and surrounding background areas, resulting in progressive optical blurring of the encoded microstructure.

**Figure 5:**
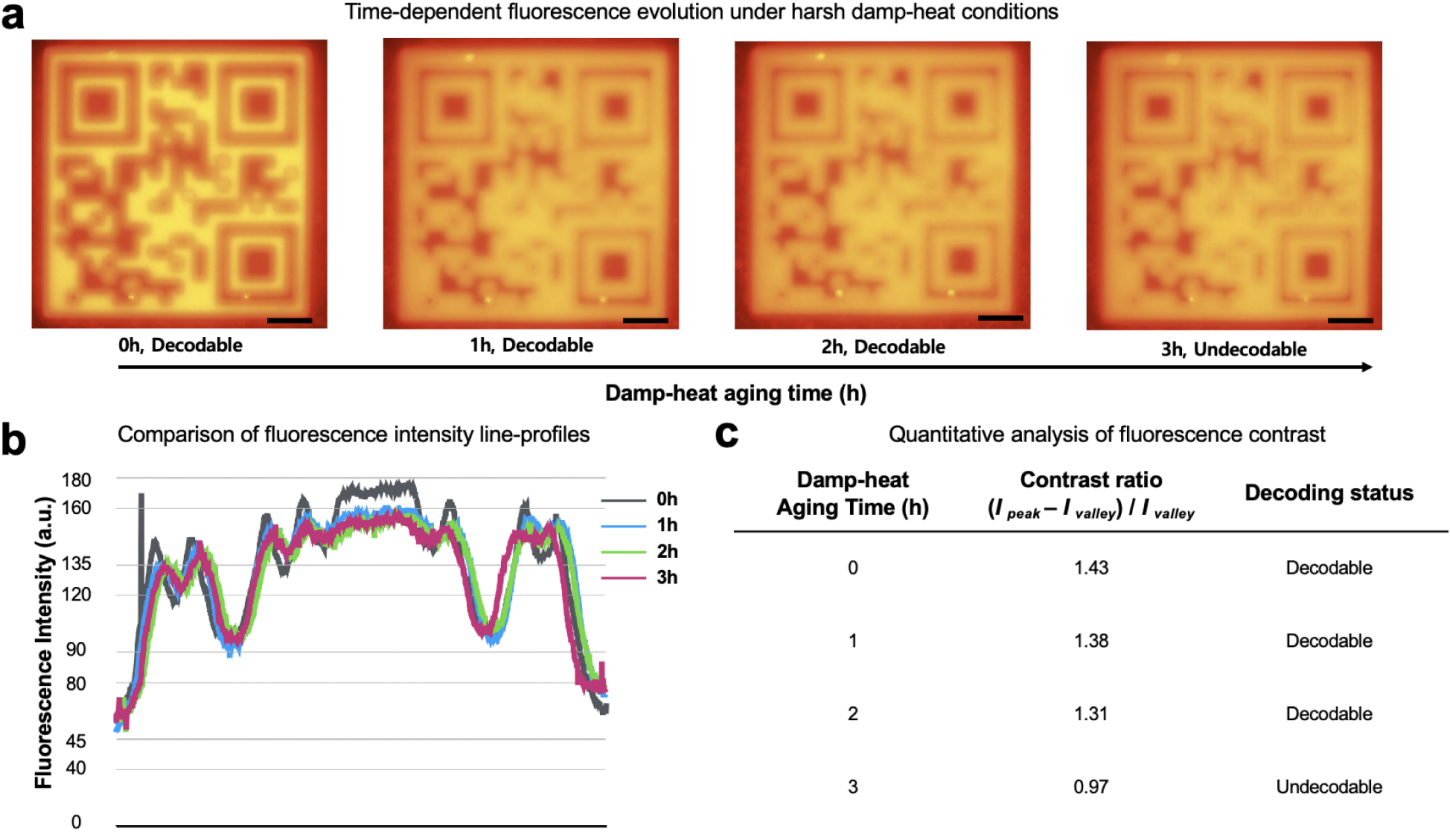
Environmental stability evaluation of hydrogel-encapsulated fluorescence QR-code structures under harsh damp-heat conditions (85 °C/85% RH). (a) Time-dependent fluorescence evolution and QR-code decoding behavior during damp-heat aging. QR-code decoding was evaluated using a default smartphone camera under identical fluorescence imaging conditions. (b) Comparison of fluorescence intensity line profiles extracted from the QR-code structures after different damp-heat aging times. (c) Quantitative analysis of fluorescence contrast ratio and QR-code decoding status during prolonged damp-heat aging. The contrast ratio was calculated as (Ipeak – Ivalley)/Ivalley, where Ipeak and Ivalley denote the maximum and minimum fluorescence intensities extracted from the line profiles, respectively. Scale bars: 100 μm.

The QR-code structures remained directly decodable using the default smartphone camera for up to 2 h under harsh damp-heat conditions, although gradual fluorescence contrast reduction and pattern blurring were observed during prolonged damp-heat aging. After 3 h of harsh damp-heat aging, reliable QR-code decoding was no longer achieved under identical fluorescence imaging and decoding conditions.

To further estimate the stability under normal ambient conditions, a rough lifetime extrapolation was performed using a simplified Peck-type temperature–humidity acceleration model.^28^ The acceleration factor was estimated using representative conservative acceleration parameters ((E_a = 0.5) eV and (n = 2)), resulting in an acceleration factor of approximately 75× between the harsh damp-heat condition (85 °C/85% RH) and typical ambient conditions (25 °C/50% RH). Based on this rough estimation, the QR-code readability maintained for up to 2 h under harsh damp-heat aging may roughly correspond to approximately 150 h (∼6 days) of operational stability under normal ambient environments. However, this estimation should be interpreted cautiously because the acceleration parameters for the present PEGDA/PUA encapsulated fluorescence QR-code system were not experimentally fitted in this study.

These results demonstrate that the hydrogel-encapsulated fluorescence QR-code structures maintained detectable fluorescence patterns and decoding capability even under severe damp-heat conditions, while also highlighting the gradual fluorescence degradation and contrast reduction that occur during prolonged environmental aging.

## Discussion

The hydrogel encapsulation strategy presented in this work demonstrates the transformation of microscale encoded structures into covert taggants that remain visually undetectable under bright-field observation while maintaining reliable fluorescence-based readability. In conventional microscale patterning systems, structural features often remain optically detectable due to refractive index contrast or surface topography differences between patterned and surrounding regions.

However, encapsulation of the fluorescent QR structure within mechanically robust PUA layers significantly suppresses optical contrast under bright-field illumination, rendering the patterned structure visually indistinguishable from the surrounding hydrogel matrix. This optical masking effect is attributed to smoothing of the optical interface and reduction of contrast generated by internal microstructures. In contrast, fluorescence imaging selectively reveals the encoded pattern because fluorescent dye molecules are spatially confined within the PEGDA network, allowing encoded information to become accessible only under predefined fluorescence excitation conditions.

Another critical factor for reliable QR decoding is the formation of well-separated island structures within the encoded pattern. During fabrication, the fluorescent PEGDA layer must form discrete modules corresponding to binary QR elements. If unintended bridges form between neighboring modules during photopolymerization, fluorescence intensity becomes spatially continuous, significantly reducing contrast between encoded and nonencoded regions. This loss of binary fluorescence contrast directly affects decoding performance. Therefore, careful control of photopolymerization conditions and optical focus is required to maintain clear island structures and reliable fluorescence distinguishability.

Environmental stability evaluation under harsh damp-heat conditions further demonstrated that the encapsulated fluorescence QR structures maintained detectable fluorescence patterns and QR-code readability for a finite duration even under severe thermal and humidity stress conditions. Although gradual fluorescence degradation, fluorescence blurring, and contrast reduction were observed during prolonged damp-heat aging, the QR-code structures remained decodable for up to 2 h under 85 °C/85% RH conditions. The observed degradation behavior is likely associated with fluorescence diffusion, optical scattering changes, and gradual reduction of fluorescence distinguishability within the encapsulated hydrogel structure. These results suggest that the encapsulation strategy not only enables optical concealment of encoded microstructures but may also contribute to maintaining fluorescence-based readability under environmentally harsh conditions.

In addition, the multilayer hydrogel architecture demonstrated wavelength-selective multispectral encoding capability, allowing independent decoding of multiple QR structures under predefined fluorescence conditions. Such wavelength-selective fluorescence decoding may provide an additional authentication layer for covert anticounterfeiting systems by restricting information accessibility to specific optical conditions.

## Conclusion

In this study, we demonstrated a hydrogel encapsulation strategy for constructing covert microscale QR taggants that are invisible under bright-field observation yet selectively readable through fluorescence imaging. Micro-QR patterns were fabricated using photocurable PEGDA containing fluorescent dyes and subsequently encapsulated within mechanically robust PUA hydrogel layers using an OFML-based photopatterning process. The encapsulation structure effectively suppressed optical contrast under bright-field conditions while maintaining strong fluorescence signals from the encoded regions, enabling reliable decoding of the embedded QR patterns.

Furthermore, multispectral encoding was achieved by incorporating different fluorescent dyes into separate hydrogel layers, allowing independent observation and decoding of multiple QR codes using wavelength-selective fluorescence filters. This multilayer hydrogel architecture provides a simple and scalable platform for covert information encoding and multiplexed taggant systems.

Environmental stability evaluation under harsh damp-heat conditions additionally demonstrated that the encapsulated fluorescence QR structures maintained fluorescence-based readability for a finite duration even under severe thermal and humidity stress conditions. Although gradual fluorescence degradation and contrast reduction were observed during prolonged damp-heat aging, the encoded QR structures remained decodable under controlled fluorescence imaging conditions, indicating the potential applicability of the proposed system for practical anticounterfeiting and authentication applications.

Finally, practical demonstrations on glass wafers and pharmaceutical tablets confirmed that the encapsulated micro-QR structures remain visually undetectable in normal observation environments while becoming readable under appropriate fluorescence conditions. These results suggest that hydrogel-based encapsulated micro-QR tags can serve as effective covert identification elements for anticounterfeiting, secure labeling, and microscale authentication applications.

## Supplementary Material



## Notes

Figure S1: scalability and optical constraints of multiplexed QR-code microparticles; Figure S2: decoding reliability under varying distance and viewing angle; Figure S3: quantitative evaluation of bright-field concealment using gray level analysis (PDF)
