# Supplementary material for: Fluorescence-Only Readable Micro-QR Codes Achieved by Hydrogel Encapsulation
Source: ACS Omega. 2026 Jun 26;11(27):40645–52. doi: 10.1021/acsomega.6c03918 (PMC13382761; doi:10.1021/acsomega.6c03918)
Supplement: Supplementary file 1 [file ao6c03918_si_001.pdf]

Fluorescence-Only Readable Micro-QR Codes Achieved by Hydrogel Encapsulation

Yongjae Song[1,2], Jaesung Park[1,2], Changhong Cao[3], Hyeli Kim[4], Dong Chan Kim[5,6],

Cheolheon Park[7], and Daewon Lee[1, 2*]*

[1]Department of Photonics and Nanoelectronics, Hanyang University, Ansan 15588, Korea

[2]BK21 FOUR ERICA-ACE Center, Hanyang University, Ansan 15588, Korea

[3]Department of Mechanical Engineering, McGill University, Montreal, Quebec H3A 0C3, Canada

[4]Bhome Gen Co., Ltd, Bucheon 14560, Korea

[5]Department of Chemical, Biological and Battery Engineering, Gachon University, Seongnam 13120, Korea

[6]Department of Semiconductor Engineering, Gachon University, Seongnam 13120, Korea

[7]Department of Electronic Engineering, Jeonbuk National University, Jeonju 54896, Korea

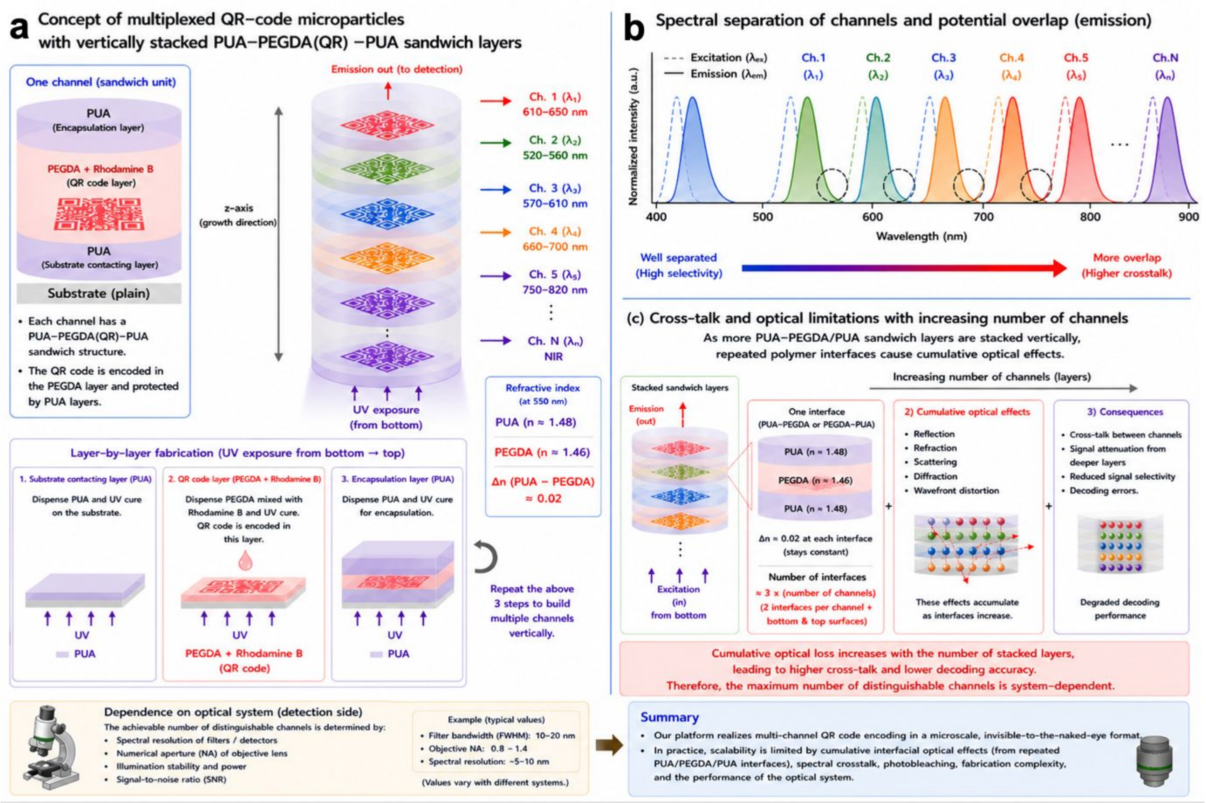

Figure S1. Scalability and optical constraints of multiplexed QR-code microparticles. (a) Schematic illustration of the layer-by-layer fabrication process for vertically stacked PUA–PEGDA(QR)–PUA sandwich structures. **(b)** Spectral separation of fluorescence channels and the potential increase in optical crosstalk as the number of channels increases. **(c)** Analysis of optical limitations in high-density multiplexing systems. Repeated refractive index mismatch ($\Delta n \approx 0.02$) at PUA/PEGDA interfaces can cumulatively induce reflection, scattering, refraction, and signal attenuation as vertically stacked channels increase. These cumulative optical effects, together with the limited depth of focus (DOF) and spectral selectivity of the objective lens, may ultimately determine the practical limit of distinguishable multiplexing channel.

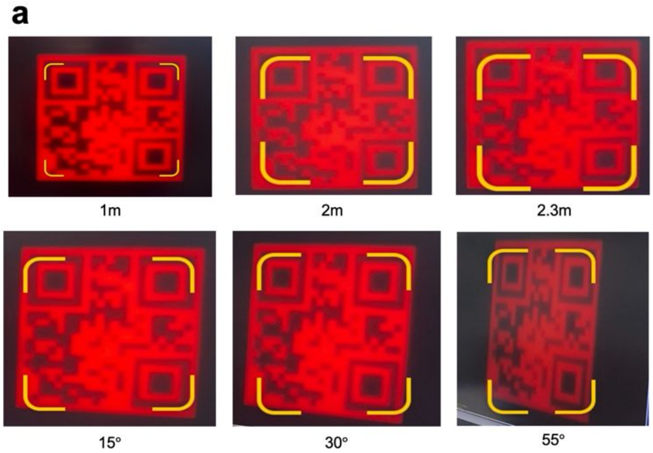

b

Distance	Trials	Success rate
1m	20	100% (20/20)
2m	20	100% (20/20)
2.3m	20	100% (20/20)
>2.3m	20	0% (0/20)
**Angle**	**Trials**	**Success rate**
15°	20	100% (20/20)
30°	20	100% (20/20)
55°	20	100% (20/20)
>55°	20	0% (0/20)

Figure S2. Decoding reliability under varying distance and viewing angle. (a) Representative decoding images at different distances (1 m, 2 m, and 2.3 m) and viewing angles (15°, 30°, and 55°). (b) Statistical evaluation of decoding success rate. Stable decoding was achieved up to 2.3 m and 55°, while decoding was not successful beyond these conditions, indicating the practical operational limits of the system.

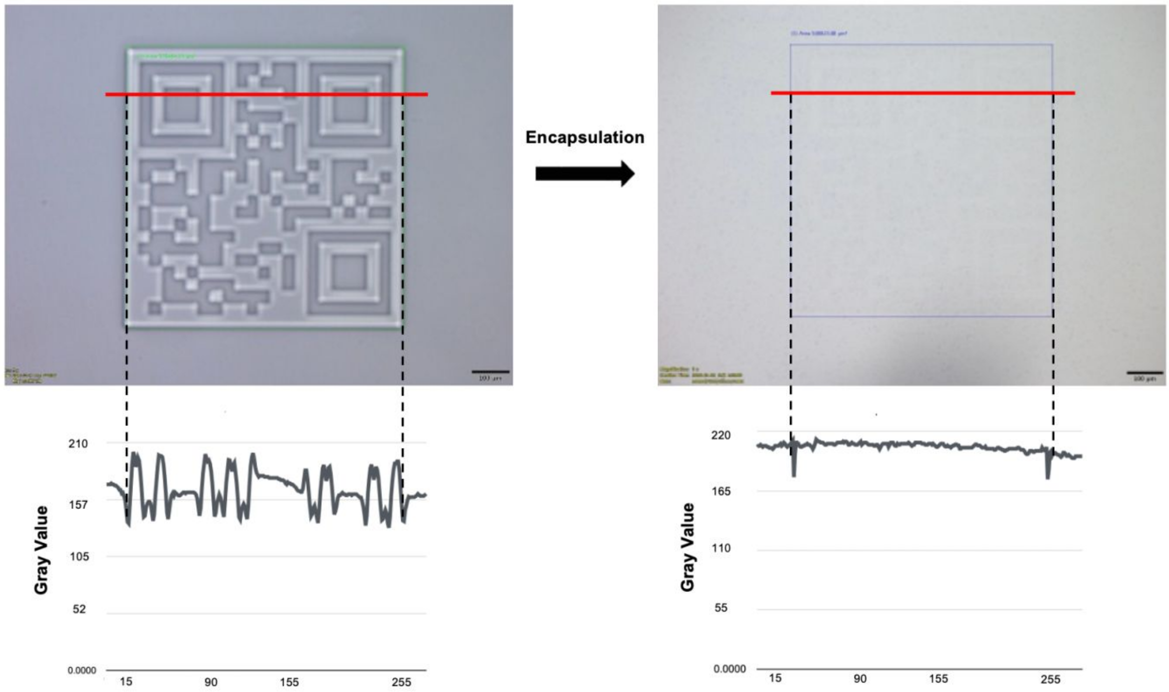

Figure S3. Quantitative evaluation of bright-field concealment using gray level analysis. Bright-field optical images of the QR-patterned structure before (left) and after encapsulation (right) are presented. The red lines indicate the regions selected for gray intensity analysis using ImageJ. The corresponding gray intensity line profiles are shown below each image. Before encapsulation, pronounced fluctuations in gray intensity are observed due to the surface topography of the QR structure, resulting in a high standard deviation ($\sigma = 15.8$). After encapsulation, the gray intensity profile becomes significantly more uniform, and the standard deviation decreases to $\sigma = 6.4$. This corresponds to an approximately 60% reduction in intensity variation, quantitatively confirming the suppression of optical contrast and the effective concealment of the embedded QR structure under bright-field conditions. Scale bar: 100 μm.
